# Fragmented Dosing of β-alanine Induces A Body Weight-Independent Pharmacokinetic Response

**DOI:** 10.3390/nu11122869

**Published:** 2019-11-23

**Authors:** Jan Stautemas, Alexia Van de Loock, Thibaux Van der Stede, Lauren Pringels, Wim Derave

**Affiliations:** Department of Movement & Sports Sciences, Ghent University, Ghent 9000, Belgium; jan.stautemas@ugent.be (J.S.); alexia.vandeloock@ugent.be (A.V.d.L.); thibaux.vanderstede@ugent.be (T.V.d.S.); Lauren.pringels@ugent.be (L.P.)

**Keywords:** food supplement, beta-alanine, carnosine, ergogenic, personalised nutrition

## Abstract

Personalised dosing of performance-enhancing food supplements is a hot topic. β-alanine is currently dosed using a fixed dose; however, evidence suggests that this might favour light compared to heavy subjects. A weight-relative dose seems to reverse this problem. In the present study, a novel dosing strategy was tested. A fragmented dose, composed of a fixed fragment of 800 mg and a weight-relative fragment of 10 mg/kg body weight, was compared to a fixed dose of 1600 mg and a weight-relative dose of 20 mg/kg body weight in a cohort of 20 subjects with a body weight ranging 48–139 kg (79.9 ± 24.4 kg). The results show that, following a fragmented dose, the influence of body weight on the pharmacokinetic response (iAUC) over a 210 min period was absent (*r* = −0.168; *p* = 0.478), in contrast to the fixed or weight-relative dose. The pharmacokinetic response also seemed more homogenous (CV% = 26%) following a fragmented dose compared to the fixed (33%) and the weight-relative dose (31%). The primary advantage of the easy-to-calculate fragmented dosing strategy is that it does not systematically favour or impair a certain weight group. Thorough dosage studies are lacking in the current field of sports and food supplements, therefore similar considerations can be made towards other (ergogenic) food supplements.

## 1. Introduction

β-alanine is a performance enhancing food supplement, shown to be effective for relatively short exercise durations, lasting between 0.5 and 10 min [1]. When supplemented chronically, it causes a rise in the dipeptide carnosine in skeletal muscle tissue, which is responsible for the ergogenic effect [2,3]. L-carnosine, constituted by the α-amino acids L-histidine and its rate-limiting factor β-alanine, is a pluripotent dipeptide, as it functions as a cellular proton buffer, an antioxidant, a metal quencher and a Ca^2+^ sensitizer [4]. In addition to the pronounced interest from the athletic community, chronic β-alanine supplementation has also been considered in, for example, elderly or soldiers [5,6].

Although higher doses have occasionally been investigated [7], the current dosing recommendation for β-alanine is to supplement a single fixed dosage of about 800–1600 mg, three or four times a day [8]. However, occasionally, weight-relative dosages have also been used [9,10,11]. Due to the lack of rationale for both fixed and weight-relative dosing strategies in previous research, we investigated whether these two strategies adequately induced a homogenous plasma response within an anthropometric diverse sample ranging from 46 to 105 kg [12]. It was shown that both dosing strategies evoke an equally large variation in pharmacokinetic response (35% and 33%, respectively) and that about 30% of the variation in physiological response could be explained by body weight. The iAUC of a fixed dose (1400 mg) was negatively correlated to body weight, indicating that heavy people had a lower physiological response compared to light people. However, following a weight-relative dose (10 mg/kg body weight (BW)), there was a positive correlation between the pharmacokinetic response and body weight. These results are in agreement with earlier data showing more side-effects in light subjects following a fixed dose, while heavy subjects perceived a higher intensity of *unusual sensation* when they ingested a weight-relative dose of 20 mg/kg BW [13]. Altogether, these results support the fact that it is not possible to homogenously supplement β-alanine in an anthropometric diverse population, with either a fixed or weight-relative dosage. This asks for a dose scaling strategy that does not systematically favour heavy or light subjects and is able to induce a more homogenous response.

Scaling is often used in drugs where a one-size-fits-all, fixed dosing, poses a risk. This is the case in drugs known to have a narrow therapeutic window and/or cause toxicity and/or where there is a clinical risk of underdosing (for example, anaesthetics or chemotherapy). β-alanine supplementation does not lead to any known damage [14]. Nevertheless, the remark has to be made that few variables have been taken into account and no research on effects exceeding 24 weeks has been undertaken. On the other hand, paraesthesia, which are considered harmless sensations of tingling and stinging, are a known side effect of acute β-alanine supplementation. At the other end of the spectrum, suboptimal dosing of β-alanine could be a relevant concern, as a dose–response curve has been established for β-alanine [15]. This indicates that carnosine loading might be suboptimal for a certain weight group when systematically favouring the heavy (weight-relative dose) or the light (fixed dose). Altogether, there seems to be a rationale to optimize β-alanine dosing.

Scaling strategies are used in drug dosing of, for example, children [16] and obese patients. Aside from body weight, other scaling factors, mostly characterized by complex calculations, have been used in medicine (for example, body surface area (BSA)) [17]. This numerical complexity could be a cause of mistakes, and it makes these scaling factors hard to translate to the general public, which would be necessary for generic nutritional supplements, such as β-alanine. Altogether, there does not seem to be a universal scaling factor, as none of these methods have been shown to be superior.

As there is no superior scaling factor, a new and easy to implement dosing strategy will be investigated in this study. We propose a fragmented dose of 800 mg + 10 mg/kg BW. The dose consists of two fragments: a fixed and a weight-relative fragment, which are set to be equal in proportion in the case of an average sized individual (e.g., 80 kg). Additionally, it partially includes body weight, which is easily measurable and does not require complex calculations. The primary aim is to investigate whether a single oral β-alanine intake, supplemented as a fragmented dose, results in a more homogenous pharmacokinetic plasma response in a sample with variable body weight, compared to the fixed and weight-relative dose. We also expect the pharmacokinetics following the fragmented dose to be independent of body weight differences.

## 2. Materials and Methods

### 2.1. Subjects

Twenty subjects (eleven male, nine female) volunteered to participate in the three testing days of the study (Table 1). Inclusion criteria were male and female, Caucasian and an age of 18–35 years. Additionally, subjects were selected to have a broad range of different body weights and we aimed to obtain a mean body weight of 80 kg in this study. Exclusion criteria were having a chronic disease, people excluded for blood donation and currently taking any food supplements or drugs (exception for contraceptives). All subjects were in good health (self-report).

The study protocol was approved by the local ethical committee (Ghent University Hospital, Belgium) and all subjects gave written informed consent in accordance with the Declaration of Helsinki.

### 2.2. Study Design

Subjects arrived fasted at the laboratory at three separate occasions and consumed a vegetarian meal the evenings prior to testing days. Upon arrival on each day, body weight and body fat percentage were measured using bio-impedance (Tanita^®^). A catheter was inserted in an antecubital vein and the first blood sample was taken using heparin coated vacutainers. Subsequently, a standardized breakfast was consumed, consisting of white bread, hazelnut paste (Nutella) and semi-skimmed milk. The calorie intake accounted for 20% of the resting metabolic rate, as calculated by the formula of Mifflin [18]. Ten minutes after the onset of breakfast, a single dose of pure β-alanine (Indis nv. Belgium) in hard gelatine capsules was ingested with water. Blood withdrawal took place every 30 min for seven occasions (30, 60, 90, 120, 150, 180 and 210 min). During the experiment, subjects abstained from any physical activity and were allowed to drink water ad libitum. The three doses of β-alanine (fixed dose: 1600 mg, weight-relative dose: 20 mg/kg BW and fragmented dose: 800 mg + 10 mg/kg BW) were allocated at random to the testing days. To obtain information about the subjective feelings and the location of paraesthesia the subjects received a standardized questionnaire in the middle and at the end of each experimental day, asking for the occurrence, intensity, localization, timing and description of possible discomfort/side-effects [19]. Gelatine capsules were manually prepared for each separate dose by an investigator involved in the investigation.

### 2.3. Blood Sample Analyses

Determination of plasma β-alanine at each time point was performed as previously published by High-Performance Liquid Chromatography-Fluorescence [12]. First, plasma samples were deproteinized using 35% sulfosalicylic acid in a 1:9 ratio. Supernatant was mixed with AccQ-tag Fluor Borate buffer and Fluor reagent from the AccQ-tag Chemistry kit (Waters sa-nv, Belgium) in a 1:7:2 ratio. Standard solutions of β-alanine were made and treated similarly prior to HPLC analysis. The samples were applied to a Waters Alliance HPLC system with the following parameters: XBridge BEH column (4.6 × 150 mm, 2.5 μm; Waters) heated to 37 °C; fluorescence detector (excitation/emission wavelength: 250/395 nm). The flow gradient used contained different amounts of buffer A (10% eluent A (Waters), 90% ddH2O), buffer B (100% acetonitrile), and buffer C (100% ddH2O) at a flow rate of 1 mL.min^-1^.

### 2.4. Pharmacokinetics and Statistical Analyses

Pharmacokinetics were investigated using a first order kinetic and non-compartmental model. Incremental area under the curve (iAUC0→210) was calculated by using the trapezoidal rule and subtracting the baseline of the AUC. Cmax was determined as the maximal concentration measured, whereas Tmax was determined as the time Cmax was reached. Elimination half-life (T1/2) was calculated as 0.693 divided by the elimination constant (Ke), whereas the Ke was computed as -2.303 multiplied by the slope of the individual linear curve of the log10 from 120 till 210 min [20]. Apparent total Clearance (Cl/F) was calculated by dividing the dose (in moles) by the iAUC, while the apparent volume of distribution (Vd/F) was calculated by dividing Cl/F by Ke. Lean body mass was determined by [total bodyweight (kg) × (1 – Fat%)], while BSA was obtained by [height(cm)^0.425^ × body weight (kg)^0.725^ × 0.007184]. The variation coefficient (CV%), standard deviation divided by mean, was determined for all variables. Before statistical analysis, normality of the continuous variables was checked using Shapiro–Wilk. Pearson correlations were performed between the anthropometric and pharmacokinetic parameters. Repeated measures MAN(C)OVA was performed to compare the pharmacokinetic response between the three different dosing strategies. In this analysis, the continuous pharmacokinetic parameters (iAUC, Cmax, T1/2, K_e_, Cl/F and Vd/F) were the different measures, with the dosing strategy being the within variable (three dosing strategies) and body weight the covariate. Tmax was analysed using the Friedman test, followed by a Wilcoxon test, where the dependent variable was Tmax (30′, 60′, 90′ or 120′) and the independent variable the different dosing strategies. To estimate the intraindividual variation following repeated dosing, a linear regression was performed where the interoccasion variability in iAUC was the dependent and the interoccasion variability in dose was the independent variable. Interoccasion variability was calculated by dividing the standard deviation in individual iAUCs or doses by the mean of the individual iAUCs or doses, respectively. All statistical analyses were carried out using the Statistical Package for the Social Sciences (version 25.0; SPSS, Chicago, IL, USA). Values are presented as mean ± SD and significance was assumed at *p* ≤ 0.05.

## 3. Results

**Figure 1:**
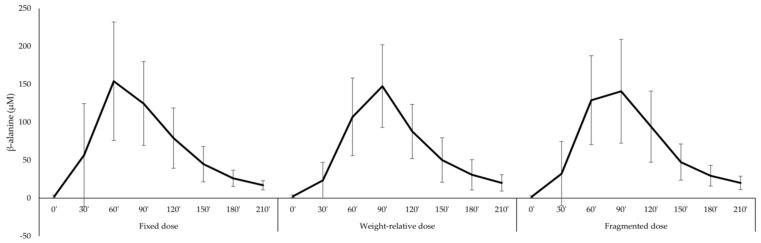
Concentration–time curve of β-alanine following three different dosing strategies.

Twenty healthy subjects, with body weight ranging from 48 kg up to 139 kg, were recruited (Table 1) to ingest three different dosages of β-alanine on three different test days. On a group level, the total β-alanine intake was similar for each dosing strategy (fixed: 1.6 g, weight-relative: 1.59 ± 0.48 g, fragmented: 1.59 ± 0.29 g; *p* = 0.999). Therefore, direct comparison between the different dosing strategies was possible. On a group level, the continuous pharmacokinetic parameters were not significantly different between dosing strategies (Figure 1; Table 2). More specifically, iAUC, a measure of the total exposure to the supplement, was similar in all three conditions. In contrast, there was a trend difference for Tmax (*p* = 0.072). Wilcoxon test revealed that Tmax following the fixed dose was lower compared to the weight-relative dose (*p* = 0.018), while there was no difference between the fragmented dose and the fixed dose or the weight-relative dose (Table 2).

**Figure 2:**
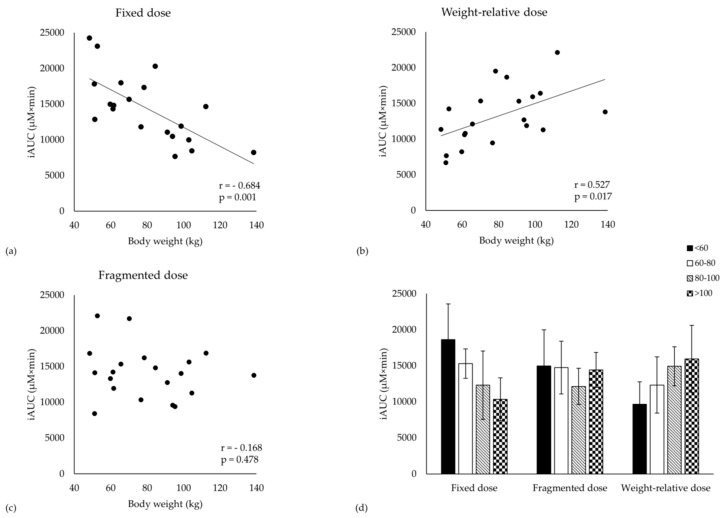
Correlations between body weight and iAUC for the (**a**) Fixed dose; (**b**) Weight-relative dose and (**c**) Fragmented dose. (**d**) iAUC shown for each dosing strategy and for body weight separated into quartiles.

The iAUC was negatively correlated to body weight when ingesting a fixed dose (*r* = - 0.684, *p* = 0.001, Figure 2a), while, following the weight-relative dosage, a positive correlation to body weight was observed (*r* = 0.527, *p* = 0.017, Figure 2b). In contrast, there was no correlation with body weight when the subjects were supplemented with the fragmented dosage (*r* = - 0.168, *p* = 0.478, Figure 2c). Similar correlations were observed between iAUC and height, BMI, resting metabolic rate, BSA and lean body mass. Body weight was able to explain 47% and 28% of the variation in iAUC following the fixed and weight-relative dosing strategy, respectively. In contrast, body weight did not explain any variance in iAUC following the fragmented dosing strategy (R² = 3%).

iAUC and Cmax are strongly positively correlated following all three dosing strategies (fixed: *r* = 0.880, weight-relative: *r* = 0.834, fragmented: *r* = 0.870; *p* < 0.001). Cmax was negatively correlated to body weight following a fixed dose, but was not correlated following the weight-relative nor the fragmented dose. Cl/F and Vd/F were positively correlated to body weight in all three dosing conditions (*r* > 0.457; *p* < 0.05).

Evidently, due to the above-mentioned correlations, body weight is a significant co-factor in the repeated measures MANOVA analyses for iAUC and Cmax (condition × body weight; *p* < 0.001). When separating the cohort into quartiles (< 60; 60–80; 80–100; > 100 kg), iAUC is 45% lower in the heavy compared to the light subjects (*p* = 0.027) following a fixed dosage (Figure 2d). On the other hand, the heavy subjects had a 65% trend higher pharmacokinetic response compared to the light subjects (*p* = 0.078) following the weight-relative dose. No differences between quartiles were found following the fragmented dose. Similar results were found for Cmax (data not shown).

**Figure 3:**
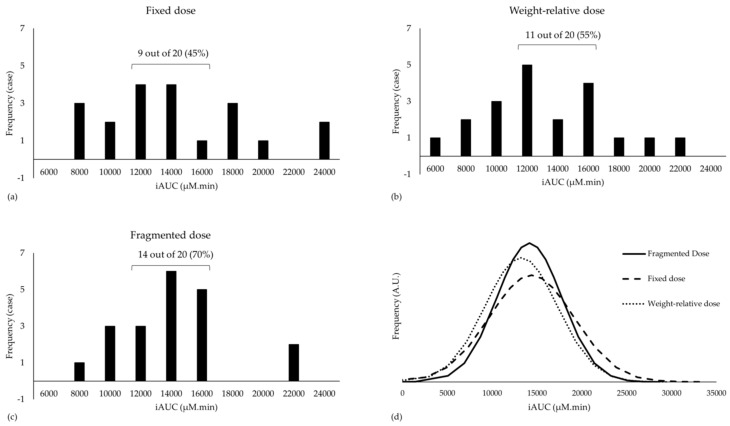
Histograms of iAUC separated for (**a**) Fixed dose; (**b**) Weight-relative dose and (**c**) Fragmented dose are shown; (**d**) normality plots of the three dosing strategies are plotted in arbitrary units (A.U.).

The CV% in iAUC when ingesting a fixed and a weight-relative dose was 33% and 31%, respectively. However, the CV% was somewhat smaller when the fragmented dose was ingested (26%). The CV% in Cmax was independent of the dosing strategy (34%–36%). This result suggests that a more homogenous plasma response is induced with a fragmented dose compared to the two other dosing strategies in our cohort. This finding is strengthened by evaluation of the histograms (Figure 3). Indeed, following the fragmented dosing strategy, 70% of our cohort had a pharmacokinetic response between 11,000 and 17,000 µM×min, which is about 20% lower or higher than the mean (Figure 3c). In contrast, only 45% and 55% had an iAUC between 11,000 and 17,000 µM×min in the fixed (Figure 3a) and the weight-relative dosing condition (Figure 3b), respectively. Similarly, the plotted normality curves based upon our data further support the statement that the fragmented dosing strategy is most likely to induce a more homogeneous plasma response (Figure 3d). Lastly, the ratio between the lowest and highest iAUC measured was 2.62 following the fragmented dose, while this was 3.16 and 3.30 for the fixed dose and weight-relative dose, respectively.

**Figure 4:**
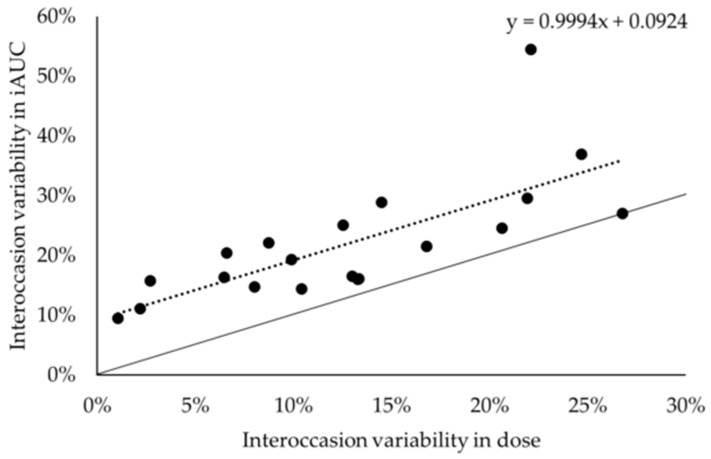
Regression between interoccasion variability in dose (x) and interoccasion variability in iAUC (y). Full line represents the line of identity, whereas the dotted line represents the regression.

The intraindividual variation is estimated to be ± 9% (Figure 4). This is based upon the regression y = 0.999 x + 9% (r² = 0.539; *p* < 0.001), where y was the interoccasion variability in iAUC and x is the interoccasion variability in dose. This suggests that when the dose would remain constant (x = 0) the variation in iAUC would be about 9%.

Symptoms of paraesthesia were reported on seven occasions, in six different subjects. Due to the low incidence of paraesthesia, only a qualitative evaluation was possible. Paraesthesia symptoms mostly seemed to be reached at Tmax, although not consistently. No relation between paraesthesia and any other parameter seemed to be present, as paraesthesia were reported (1) on test days that did and did not contain the highest individual dose of the subjects, (2) in light and heavy subjects and (3) in both sexes.

## 4. Discussion

The primary aim of this experiment was to test if a more homogenous pharmacokinetic response, independent of body weight, could be observed following the newly proposed, fragmented dosing strategy of 800 mg + 10 mg/kg BW β-alanine. This was compared to two more traditional approaches with the same total dose at the group average level: a fixed dose of 1600 mg and a weight-relative dose of 20 mg/kg BW. This question was investigated in a randomised cross-over design in a cohort of 20 healthy subjects, having a body weight ranging from 48 to 139 kg.

First of all, it was confirmed that there was a negative correlation between iAUC and body weight when a fixed dose of β-alanine was ingested by the anthropometric diverse subjects. This confirms that light subjects are most likely favoured compared to heavy people when β-alanine is supplemented as a fixed dose. On the other hand, there was a positive correlation between iAUC and body weight when a weight-relative dose of 20 mg/kg dose was ingested. This confirms that the body weight correction is not able to eliminate the inequality induced by a fixed dose. It rather reverses the problem. These results are in agreement with earlier data [12,13]. Body weight correction did also not improve homogeneity in β-alanine plasma response, compared to the fixed dose. The variation in iAUC in both conditions (fixed dose: 33%, and weight-relative dose: 31%) was comparable to earlier reports using both pure and slow release β-alanine (35%, 33% and 32%) [12,19]. Indeed, both dosing strategies evoke an equally high CV% in pharmacokinetic plasma profile and both dosing strategies do not guarantee an equally high physiological response in heavy or light subjects.

In order to decrease the observed variability in pharmacokinetic response and to eliminate the dependency on body weight, a novel dosing strategy called a fragmented dose of 800 mg + 10 mg/kg BW was proposed. The most important observation of this study is that there was no longer a correlation between iAUC and body weight when this fragmented dose was ingested by the anthropometric diverse population. Additionally, the CV% of iAUC following this dose was somewhat lower compared to the fixed and weight-relative dose (26% vs. 33% and 31%). Therefore, it can be concluded that, compared to a fixed or a weight-relative dosing strategy, a fragmented dosage elicits a more homogenous pharmacokinetics response that is independent of body weight in an anthropometric diverse sample.

From a pharmacokinetic point of view, the results presented here are not surprising. Vd/F was unchanged and positively correlated to body weight following the different dosages. Therefore, it could be stated that, for β-alanine, Vd/F is (partially) proportional to bodyweight. It therefore also stands to reason to correct the dose partially for body weight in order to obtain a response (iAUC) that is independent of body weight.

Following the fragmented dose, the CV% of iAUC was still 26%. This CV% consists primarily of interindividual variation, but also of intraindividual variation, analytical variation and random error. It could be argued that the remaining variation is caused by the heterogeneous sample and the heterogeneity in dosages (1280–2185 mg). However, when looking at the three subjects that received more or less the same dose on three different occasions (weighing 77, 78 and 84 kg), it can be observed that the CV% was 26% as well. Conclusions based upon this small, but homogenous, subsample of three subjects, need to be taken with caution. However, they seem to suggest that the remaining 26% of variation represents variation that is not caused by the heterogeneity of the sample. The origin of the remaining variation warrants further investigation and could contain targets for other personalised measures (e.g., nutrigenetics of β-alanine transporters).

It also remains to be elucidated if the different dosing strategies affect Tmax. This is suggested by our data; however, caution is warranted as Tmax was a categorical and not a continuous variable. In each condition, most subjects (> 17) had a Tmax of either 60′ or 90′, which might indicate that in these subjects the actual Tmax is somewhere between 60′ or 90′. Therefore, it is not unlikely that actual difference between conditions is smaller than reported. Additionally, it is unclear how a change in Tmax would functionally impact side-effects or carnosine loading.

Based upon the results of our healthy but diverse population, we do not expect that other anthropometric scaling factors would have been better alternatives. A first argument is that in the current population, the anthropometric parameters body weight, height, BSA, lean body mass, BMI and resting metabolic rate are strongly correlated to each other (*r* > 0.900). Therefore, there is no correlation between these anthropometric parameters and the iAUC of the fragmented dose, while there is a negative/positive correlation in the case of the fixed/weight-relative dose, respectively. Secondly, the current dose is much easier to calculate compared to these other scaling factors and therefore relatively easy to implement.

When considering scaling in medicine, pharmacodynamics or clinical outcomes are mostly taken into account. Unfortunately, at this point there is no study linking the acute or chronic plasma β-alanine profile to chronic changes in carnosine and ergogenic response. However, we hypothesize that iAUC is presumably the most important measure, as iAUC represents the concentration over time above baseline levels. The current dogma is that the increase of carnosine in muscle depends on the bioavailability of β-alanine in muscle [8]. β-alanine is mainly transported into muscle by the taurine transporter, TauT. The Km of TauT is about 40–46 µM [21,22], which is relevantly higher than homeostatic levels of β-alanine (2.27 ± 1.60 µM). Although the physiological endpoint is not considered yet, iAUC represents the concentration above baseline over time and is therefore a good measure. Relevantly, it has also been shown that the acute iAUC is a stable pharmacokinetic parameter following chronic β-alanine supplementation (3×10 mg/kw BW – 15 days) [2].

Chronic β-alanine supplementation has been and is being used in a variety of anthropometric diverse subjects, for example, rugby union athletes (102.6 ± 11.6 kg) [23] and COPD patients (48.9 – 95.0 kg; 71.9 ± 13.7 kg) [24]. To account for the variety in subjects and for the inequality induced by a fixed dose, the fragmented dose could be a useful alternative to determine the daily dose of β-alanine in a supplementation protocol. We would hypothesize that a daily dose of three or four times 800 mg + 10 mg/kg BW could be suitable (Table 3). A dose–response relation has been established for the ingestion of β-alanine [15,25]. This implies that, independent of body weight, an increase in carnosine and potentially ergogenic effect could be expected when more β-alanine is ingested. However, when body weight is taken into account, it can be hypothesized that, following chronic fixed dosing, light subjects are also favoured. Indeed, a negative correlation between body weight and the change in carnosine following chronic fixed dose β-alanine supplementation has already been observed in the gastrocnemius muscle of male subjects [26].

In practice, commercial β-alanine is available in a wide variety of single dosages of 0.5 g up to 1.35 g, but it can also be acquired as powder. With the current evidence in mind, one should consider that the current recommendations were made based upon the mean (± 70–80 kg) and that subjects weighing less or more could perform dose adjustments by using the fragmented dosage (Table 3). Additionally, the fragmented dose might be very relevant for scientists, as, with the fixed or weight-relative dosing strategies, non-random error might be introduced to the data, which could cause bias. When using a homogenous sample, implications are limited. However, in a heterogeneous sample, the concerns raised in this discussion are highly relevant.

Personalised dosing is a promising line of investigation that is currently receiving increased attention [27]. The first successful nutrigenetic study in the field of sports nutrition was recently published [28], which might further surge attention to this topic. We believe that it indeed is worthwhile to investigate these more complex, expensive and harder to implement personalised dosing strategies. However, in our opinion, the current dosing practices of most ergogenic food supplements could, similarly to the current investigation, first be re-evaluated, in order to detect easier measures for dose adaptations.

It is important to consider that this investigation is performed in a mixed-sex design, using only healthy young Caucasian subjects. Therefore, generalisation to other groups needs to be performed with caution. Urine was not collected in this study, limiting conclusions about the bioavailability. However, urinary excretion of β-alanine is known to be low (< 5%) [2]. The current investigation is also performed using pure β-alanine, possibly limiting generalisation towards slow release β-alanine. However, the concentration-time curve and the lack of severe paraesthesia clearly suggest a pharmacokinetic profile similar to slow release β-alanine. This is most likely due to the ingestion of a small breakfast 10 min before β-alanine intake, which might be able to affect absorption kinetics through changed gastric emptying [29]. As β-alanine is supplemented throughout the day, it is seldom ingested in a fasted state. Additionally, co-ingestion of β-alanine with a meal could be part of the current recommendations [30]. Altogether, the results represented here are most likely transferable to practice.

## 5. Conclusions

The current study shows that when β-alanine is ingested in a fragmented dose, composed of a fixed and a weight-relative portion, the effect of body weight on the pharmacokinetic response is obliterated. Secondly, the pharmacokinetic response was more homogenous with a fragmented dosing strategy, compared to the commonly used fixed dose and the weight-relative dose.

## Figures and Tables

**Table 1:** Anthropometric characteristics.

		Female	Male	All
	*n*	9	11	20
Age	year	23.3 ± 4.1	23.7 ± 2.3	23.6 ± 3.1
*Min–Max*				*21–34*
Body Weight	kg	62.9 ± 15.4	93.8 ± 21.7	79.9 ± 24.4
*Min–Max*				*48.3–138.5*
Height	m	1.71 ± 0.08	1.87 ± 0.08	1.80 ± 0.11
*Min–Max*				*1.64–2.05*
Fat %		26.3 ± 9.3	20.0 ± 7.00	22.9 ± 8.5
*Min–Max*				*7.8–46.2*
Lean Body Mass	kg	73.7 ± 11.3	45.1 ± 4.4	60.8 ± 17.0
*Min–Max*				*39.9–95.8*
Body Mass Index	kg.m^-2^	21.6 ± 5.1	26.5 ± 4.6	24.3 ± 5.3
*Min–Max*				*16.7–34.2*

Mean ± SD are shown, unless otherwise stated.

**Table 2:** Pharmacokinetic parameters.

		Fixed Dose	Weight-Relative Dose	Fragmented Dose	Multivariate *p*
Total Dose	g	1.6	1.59 ± 0.48	1.59 ± 0.29	0.999
Incremental Area Under the Curve	µM×min	14391 ± 4722	13232 ± 4054	14148 ± 3630	0.524
Maximal Concentration	µM	179 ± 65	155 ± 53	168 ± 59	0.298
Time of Maximal Concentration	min	72.0 ± 22.6^a^	87.0 ± 16.6^b^	82.5 ± 21.5^a,b^	0.072
Elimination Rate Constant	1/h	0.983 ± 0.227	0.998 ± 0.158	0.991 ± 0.298	0.972
Elimination Half-Life	h	0.748 ± 0.205	0.712 ± 0.124	0.760 ± 0.222	0.667
Apparent Total Clearance	l/h	83.0 ± 27.8	84.5 ± 23.5	81.2 ± 24.3	0.671
Apparent Volume of Distribution	l	90.6 ± 40.5	88.9 ± 36.8	92.8 ± 44.9	0.873

Mean ± SD are shown. Different letters indicate statistical difference between dosages (*p* < 0.05).

**Table 3:** Fragmented dosing scheme (800 mg + 10 mg/kg body weight).

Body Weight	Daily Dose (g) = 3–4 Times Single Dose	Single Dose (g)
40 kg	3.6–4.8	1.2
60 kg	4.2–5.6	1.4
80 kg	4.8–6.4	1.6
100 kg	5.4–7.2	1.8
120 kg	6.0–8.0	2.0
140 kg	6.6–8.8	2.2
